# The Antioxidant Enzyme Methionine Sulfoxide Reductase A (MsrA) Interacts with Jab1/CSN5 and Regulates Its Function

**DOI:** 10.3390/antiox9050452

**Published:** 2020-05-24

**Authors:** Beichen Jiang, Zachary Adams, Shannon Moonah, Honglian Shi, Julie Maupin-Furlow, Jackob Moskovitz

**Affiliations:** 1 Department of Pharmacology and Toxicology, School of Pharmacy, University of Kansas, Lawrence, KS 66045, USA; jiangbeichen@hotmail.com (B.J.); hshi@ku.edu (H.S.); 2 Department of Microbiology and Cell Science, College of Agricultural and Life Sciences, University of Florida, Gainesville, FL 32611, USA; zachadams@ufl.edu (Z.A.); jmaupin@ufl.edu (J.M.-F.); 3 Division of Infectious Diseases, University of Virginia, 345 Crispell Drive, MR-6, Room 2715, Charlottesville, VA 22908, USA; SM5FE@hscmail.mcc.virginia.edu

**Keywords:** methionine oxidation, posttranslational modification, neddylation, ubiquitin, brain, oxidative stress

## Abstract

Methionine sulfoxide (MetO) is an oxidative posttranslational modification that primarily occurs under oxidative stress conditions, leading to alteration of protein structure and function. This modification is regulated by MetO reduction through the evolutionarily conserved methionine sulfoxide reductase (Msr) system. The Msr type A enzyme (MsrA) plays an important role as a cellular antioxidant and promotes cell survival. The ubiquitin- (Ub) like neddylation pathway, which is controlled by the c-Jun activation domain-binding protein-1 (Jab1), also affects cell survival. Jab1 negatively regulates expression of the cell cycle inhibitor cyclin-dependent kinase inhibitor 1B (P27) through binding and targeting P27 for ubiquitination and degradation. Here we report the finding that MsrA interacts with Jab1 and enhances Jab1′s deneddylase activity (removal of Nedd8). In turn, an increase is observed in the level of deneddylated Cullin-1 (Cul-1, a component of E3 Ub ligase complexes). Furthermore, the action of MsrA increases the binding affinity of Jab1 to P27, while MsrA ablation causes a dramatic increase in P27 expression. Thus, an interaction between MsrA and Jab1 is proposed to have a positive effect on the function of Jab1 and to serve as a means to regulate cellular resistance to oxidative stress and to enhance cell survival.

## 1. Introduction

The methionine sulfoxide reductase (Msr) system consists of Msr type A (MsrA) and Msr type B (MsrB) that reduce S-MetO and R-MetO, respectively [1,2]. This basic system is highly conserved in evolution and is designed to “repair” oxidatively damaged proteins containing MetO residues [1,2]. Methionine oxidation is a common phenomenon that can alter protein function through allosteric changes of a protein [1], reduce or increase protein stability by changing its hydrophobic properties [1], and regulate protein function via changing the redox status of a pivotal Met residue [1]. A bioinformatics tool is recently developed to model the contribution of MetO residues to protein structure and function, strengthening knowledge of how global and important this modification is to biological processes [1]. A compromised expression or activity of MsrA is found to lead to various cellular malfunctions. The disruption of the msrA gene in *Saccharomyces cerevisiae* causes hypersensitivity to oxidative stress [3]. Likewise, MsrA knockout (KO) mice are more vulnerable to oxidative stress and demonstrate several molecular phenotypes that can be linked to age-associated diseases when compared to wild type (WT) [4]. For example, MsrA KO mice exhibit many of the neuropathological traits associated with Alzheimer’s disease (AD) [5] and Parkinson’s disease (PD) [6,7,8]. The crossed MsrA KO x AD model showed stronger phenotypes with respect to mitochondrial malfunction and the distribution of beta-amyloid forms compared with the AD model [9]. A compromise in MsrA activity can cause other organ or cellular malfunctions that are not directly linked to neurodegeneration. These include, for example, mental health disorders [8], heart disease [10], liver toxicity [11], and cancer [12]. Additionally, MsrA is involved in maintaining the basic cochlea structure of the inner ear, and its deficiency may contribute to hearing loss [13]. We also find MsrA regulates the Ub-like modification of proteins in *Archaea* and the ubiquitination of 14-3-3 ζ in a mouse brain [14,15], suggesting a deep evolutionary association of MsrA with Ub/Ub-like systems.

**Figure 1:**
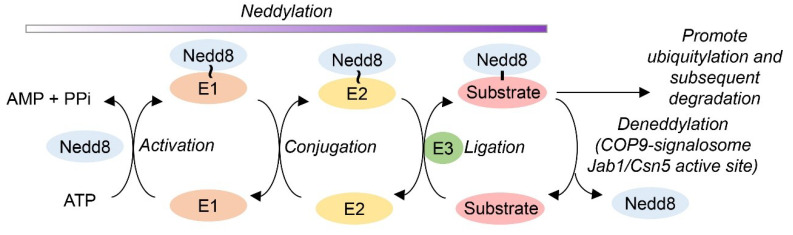
Post-translational modification of proteins by neddylation. The ubiquitin-like protein Nedd8 is covalently bound to substrate proteins by a cascade of E1 activating, E2 conjugating and E3 ligase enzymes through a process termed neddylation. Deneddylation regulates this process and is catalyzed by the c-Jun activation domain-binding protein-1 (Jab1)/Csn5 subunit active site of the COP9-signalosome, which cleaves the isopeptide linkage to release neural precursor cell expressed developmentally down-regulated 8 (Nedd8) from the substrate protein. ~, thioester intermediate. —, isopeptide linkage.

Neddylation is a posttranslational modification system that adds the ubiquitin-like neural precursor cell expressed developmentally down-regulated 8 (Nedd8) to substrate proteins [16] (Figure 1). Nedd8 is covalently ligated to a limited number of cellular proteins in a manner analogous to ubiquitination. In a canonical neddylation process, Nedd8 is activated by the Nedd8 activating enzyme (NAE) [17]. Nedd8 is then transferred from the NAE via the Nedd8 conjugating enzyme (NCE) and the RING-box protein RBX1 to the Cullin subunit of Cullin/RING ubiquitin ligases (CRL) [18]. RBX1 serves as the E3 ligase for Nedd8 and as an E3 ligase for subsequent ubiquitination reactions [19]. The Cullin subunits of CRLs are the best-studied neddylation substrates. Neddylation loosens the interaction of RBX1 with the WHB domain and RBX1 can subsequently promote E2-dependent ubiquitination and protein degradation [20]. CRLs are the largest family of multisubunit E3 ubiquitin ligases, controlling the degradation of about 20% of the proteasome-regulated proteins that are involved in many aspects of important biological processes [21,22,23]. Removal of Nedd8 from proteins is mediated by c-Jun activation domain-binding protein-1 (Jab1) (synonym CSN5), which is the fifth subunit of the constitutive photomorphogenic-9 signalosome (COP9). Jab1 was initially identified as c-Jun activation domain-binding protein-1, hence the nomenclature [24]. The COP9 signalosome (CSN) is evolutionarily conserved among all eukaryotes and has a canonical composition of eight subunits (CSN1–8) found in all multicellular organisms. CSN regulates the activity of the CRLs, the largest family of ubiquitin E3 ligases. Regulation of CRLs by the CSN involves the removal of Nedd8 from Cul-1, the cullin scaffold subunit of CRLs, through the hydrolytic activity of a metalloprotease MPN+/JAMM motif (the c-Jun binding domain) within the catalytic Jab1 subunit of CSN. In short, CSN promotes deneddylation of Cul-1, and Jab1 provides the catalytic center to execute this isopeptidase activity [25,26,27,28]. Interestingly, although Jab1 only exhibits deneddylase activity when it interacts with the other CSN components [29,30], a large portion of the free Jab1 is detected in both cytoplasm and nucleus [31] suggesting Jab1 may have a CSN-independent function. The deneddylation of Cul-1 by the Jab1 active site of CSN acts as an upstream regulator of Skp1/Cul-1/F-box (SCF)-dependent ubiquitination of numerous substrates, including P27 and IκBα [32]. P27 is a universal cyclin-dependent kinase (CDK) inhibitor that directly inhibits the enzymatic activity of cyclin-CDK complexes, resulting in cell cycle arrest at G1 [33]. Jab1 promotes cell proliferation and inactivates P27 by inducing translocation of P27 from the nucleus to the cytoplasm, which accelerates P27 degradation through the Ub-dependent proteasome pathway and promotes cell cycle progression [34]. Thus, although transcriptional regulation is possible, the cellular expression of P27 is primarily regulated at the posttranslational level by Jab1 and by the Ub-proteasome pathway [34].

Here we show that MsrA interacts with Jab1 and consequently, the Jab1 deneddylase activity (removal of Nedd8) is enhanced, causing an increased deneddylation of Cul-1. Furthermore, the action of MsrA enhances the binding affinity of Jab1 to P27, causing a reduction in P27 level (presumably through its degradation by the Ub system). This novel character of MsrA as a positive regulator of Jab1 function will have implications for the general role of MsrA in mediating protein ubiquitination / neddylation and regulation of cell survival.

## 2. Material and Methods

### 2.1. Ethics Statement

The use of wild-type (WT) and MsrA knockout (KO, MsrA^−^/^−^) mice were followed according to an approved Animal protocol by the Institutional Animal Care and Use Committee (IACUC) of the University of Kansas. The protocol was implemented in conjunction with the Service Policy (PHS) and Animal Welfare Act (AWA) for the humane care and use of laboratory animals. The identification code of IACUC protocol is 144-01 and the Animal Welfare Assurance Number is A3339-01. The approval date of the committee was 7 July 2018.

### 2.2. The Yeast Two-Hybrid System (Y2H)

A yeast Activation Domain (AD) two-hybrid (Y2H) library (“prey”) was screened in a haploid strain by mating with the opposite mating strain expressing the DNA binding domain (BD)-bait fusion protein (“bait”). Interaction of the BD-bait and AD-prey proteins was assessed based on the expression of the reporter genes in conditional autotrophs; this approach allowed capture of the AD plasmid(s) expressing the interacting proteins. The AD library and BD-bait were transformed into the yeast strain, Y187, and Y2HGold, respectively. The Y2HGold strain with full-length MsrA as bait was screened against an adult human brain cDNA library (Clontech, Mountain View, CA, USA) by Next Interactions Inc. (Richmond, CA, USA). The Y2H procedures were performed similarly to Clontech’s protocol of the Matchmaker Gold Yeast Two-Hybrid System. Constructs were made using Clontech’s pGBKT7 and pGADT7 plasmids that are components of the Matchmaker Gold Yeast Two-Hybrid System. The MsrA clone was purchased from DNASU Plasmid Repository (The Biodesign Institute, Tempe, AZ, USA). Constructs encoding the MsrA protein in pGBKT7 (“bait”, Trp1 selection marker of Y2H Gold yeast strain) and pGADT7 (“prey”, Leu2 election marker, Y187 yeast strain) were mated using the following procedure. An YPD-agar plate was used to streak the *S. cerevisiae* strains and then the strains were incubated in YPD medium overnight at 30 °C with rotation at 225 rpm. The *S. cerevisiae*/YPD culture was diluted 1:10 with fresh YPD medium and incubated for an additional 2 h at 30 °C with rotation (cell density: between 10^7^ and 3.0 × 10^7^ cells per mL). The cells were centrifuged at 580× *g* and the pelleted cells were resuspended in YPD medium and streaked onto agar plates, containing the standard selective media (SD) lacking tryptophan (Trp) and leucine (Leu). The plates were incubated for five days at 30 °C. Then, colonies from each plate were resuspended in H_2_O and spotted onto agar plates lacking Trp and Leu or agar plates containing selective media lacking Trp, Leu, and histidine (His). Selection for positive clones was done for HIS3 reporter activation [low stringency, SD-Leu,-Trp, and -His (LTH)] and for HIS3 and ADE2 reporter activation [high stringency, SD-Leu, -Trp, -His, and -Adenine (LTHA)]. The initial selection was performed for 5 days. The equivalent of 1.07 × 10^6^ diploid cells was selected on SD-LTH and the equivalent of 7.4 × 10^6^ on SD-LTHA. Isolated colonies were picked and re-streaked for 2- to 3-day growth on high stringency medium (SD-LTHA) to ensure robust growth. Fifty-three colonies were finally obtained and prey cDNAs were amplified with colony PCR using a set of flanking primers. The resulting PCR products were sequenced in the forward direction as follows. Colonies from SD-LTH and SD-LTHA agarose plates were picked into a grid to grow larger quantities of cells. After regrowth, each colony was transferred into a 20 mM NaOH suspension and then boiled for 20 min. After cooling, lysates were clarified by centrifugation at >3000× *g* for 5 min. The supernatants were amplified by PCR using Mango Polymerase buffer, nucleotides mix, primers [NIXO-1838 (5′-gatGAAGATACCCCACCAAACC-3′) and NIXO-1839 (5′-acgatgcacagttgaAGTGAA-3′)], Mango Tag DNA polymerase (Bioline, London, UK), and HiFi Tag DNA polymerase (Clontech, Mountain View, CA, USA). Aliquots from each amplified sample were analyzed for DNA bands by electrophoresis using a 1% (*w/v*) TAE agarose gel and sequenced. DNA sequences were compared to the human proteome database (UniProtKB). Hits that did not map to the coding sequence of known protein sequences or were of bad quality were not analyzed further.

### 2.3. GST-MsrA “Pull-Down” Experiments

Brain extracts of WT and MsrA KO mice (*n* = 3 per strain, 6 months of age) were made by homogenization of postmortem brains in PBS in the presence of protease inhibitor cocktail (Sigma-Aldrich, St. Louis, MO, USA) on ice. The insoluble fraction of each extract was removed by high-speed centrifugation (10,000 *g* for 10 min). The supernatants were collected, and protein concentration of each preparation was determined using the Bradford reagent and BSA as the protein standard (Bio-Rad, Hercules, CA, USA). The MsrA KO supernatant (20 µg protein) was incubated with Sepharose glutathione-*S*-transferase (GST)-MsrA or Sepharose-GST resin, serving as control. The resins were synthesized as previously described with the MsrA derived from bovine liver cDNA expressed in *Escherichia coli* [35]. The incubation of the resins with the lysates was carried out for two hours at room temperature followed by overnight incubation at 4 °C. Thereafter, the resins were washed with PBS by multiple low-speed centrifugations until no protein was detected in the washing buffer (PBS). The proteins bound to the washed resins were eluted by 2× reducing SDS-gel-electrophoresis buffer, boiled, and separated by 4–20% reducing SDS-gel-electrophoresis. One lane containing the GST-MsrA protein was stained along with the molecular standards for protein with Coomassie Blue R-250 stain. Additional gel-separated protein lanes were subjected to Western blot analysis using anti-Jab1 antibody (1:1000 dilution) as the primary antibody (Thermo-Fisher Scientific, Waltham, MA, USA), and the appropriate secondary HRP-conjugated antibody (Bio-Rad, Hercules, CA, USA).

### 2.4. Immunoprecipitation (IP) Experiments

Antibodies used included: anti-MsrA antibody (Proteintech Group, Rosemont, IL, USA), anti-Jab1 antibody (Thermo-Fisher Scientific, Waltham, MA, USA), anti-Cul-1 antibody (Novus, Littleton, CO, USA), anti-Nedd8 antibody (Boster Bio, Pleasanton, CA, USA), anti-P27 antibody (Proteintech Group, Rosemont, IL, USA), anti-β actin antibody (Abcam, Cambridge, UK), and HRP-conjugated secondary antibodies for Western blot analyses (Rabbit anti-mouse and Goat anti-rabbit) (Bio-Rad, Hercules, CA, USA). WT and MsrA KO mouse brain supernatants were incubated with anti-MsrA antibody (Proteintech Group, Rosemont, IL, USA) in the presence of protein-G Sepharose at room temperature for the first two hours, followed by overnight incubation at 4 °C. Thereafter, the resins were washed with PBS by multiple low-speed centrifugations until no protein was detected in the washing buffer (PBS). The proteins that bound to the washed resins were eluted and subjected to Western blot analysis as earlier described.

### 2.5. In Vivo Met Oxidation of Jab1 in Mouse Brain

Six-month-old WT mice (*n* = 5 males) were exposed to normoxia (20% oxygen) or max hyperoxia (100% oxygen) for 24 h, as previously described [4]. The max hyperoxia conditions foster the formation of MetO while ensuring the welfare of the animals [4]. Thereafter, the mice were euthanized and brain extracts were made in PBS, as previously described [4]. Immunoprecipitation with rabbit anti-MetO antibody [36] was performed using 500 µg of protein extracted from each brain, followed by Western blot analysis probed with the mouse primary anti-Jab1 antibody (Thermo-Fisher Scientific, Waltham, MA, USA).

### 2.6. Deneddylase Activity of Jab1 on Cul-1

The in vivo deneddylation activity of Jab1 on its substrate (Cul-1) was determined as a function of the presence or absence of MsrA in mouse brain and liver extracts. To achieve this goal, IP experiments were performed using the anti-Cul-1 antibody (Novus, Littleton, CO, USA) as the IP antibody and the anti-Nedd8 (Boster Bio, Pleasanton, CA, USA) as the primary antibody in the subsequent Western blot analysis and vice-versa. Furthermore, based on the supposition that Jab1 would bind to the Nedd8 moiety of Cul-1 or other neddylated proteins, an anti-Jab1 antibody was used in an IP experiment. In this IP experiment, the anti-Jab1 antibody was used as a “fishing” probe to isolate neddylated protein complexes, such as Cul-1, that may be bound to Jab1 from biological extracts (prior to the removal of Nedd8 by Jab1 and its associated cofactors). These anti-Jab1 immunoprecipitants were thereafter probed by Western blot analysis using anti-Cul-1 antibody.

### 2.7. Recombinant Nedd8 (FHN8) Conjugate Synthesis and Purification

An N-terminal tandem Flag-His_6_-tag was added to the coding sequence of the mature Nedd8 protein (Uniprot accession P29595, residues 1-76), with the removal of the original initiator methionine. The recombinant coding sequence was codon-optimized for translation in *Haloferax volcanii* using the Codon Optimization On-Line (COOL) web platform. NdeI/BlpI restriction sites were added to the sequence 5′ and 3′ ends, and the entire sequence was synthesized by GenScript and ligated into pUC57 plasmid to create a pJAM3518 plasmid. Flag-His_6_-Nedd8 was removed from the pJAM3518 by NdeI/BlpI digestion and then ligated into a pJAM202 plasmid to create the pJAM3519 plasmid. Finally, the pJAM3519 was transformed into an *E. coli* TOP10, and isolated transformants were patched on LB supplemented with ampicillin. PCR screening by primers HvFW/RV confirmed the presence of the plasmid and the correct size of the FLAG-His_6_-Nedd8 (FHN8) insert. Several patches were selected, and the integrity of the insert sequence was verified by Sanger DNA sequencing. Plasmid pJAM3519 was passaged through a methylation deficient strain of *E. coli* (GM2163) prior to transformation into *H. volcanii.* Note that several strains of *H. volcanii* expressing FHN8 were tested for conjugate formation at a small scale; HM1096 was used as it produced the highest levels observed. Accordingly, *H. volcanii* HM1096 cells expressing pJAM3519 were inoculated in ATCC974 medium, supplemented with 50 mM DMSO and 0.2 µg/mL novobiocin. Flasks were incubated at 42 °C for 93 h with rotary shaking at 200 rpm. Cultures were pooled and centrifuged at 4000× *g* for 15 min and resuspended in TBS-2M (2M NaCl, 50 mM Tris-Cl, pH 7.4) supplemented with protease inhibitor cocktail (Thermo-Fisher Scientific, Waltham, MA, USA). Thereafter, the cell suspension was thrice passed through a French pressure cell (Laboratory Supply Network, Atkinson, NH, USA) (2000 psi). The resulting lysate was supplemented with imidazole [30 mM] and clarified by centrifugation (13,000× *g*). Clarified supernatant was applied to a 1 mL HisTrap HP column equilibrated in TBS-2M supplemented with 30 mM imidazole. After loading of the lysate, the column was extensively washed in the same buffer and the column-bound protein was eluted using TBS-2M supplemented with 500 mM imidazole. Peak fractions were supplemented with 1 mM EDTA and analyzed in a reducing 4–20% SDS-PAGE followed by subsequent Coomassie Blue R-250 staining and analysis by Western blotting. Selected fractions were pooled and dialyzed at 4 °C against TBS-2M.

### 2.8. Deneddylation Activity Assay

Equal amounts of protein (500 μg) extracted from the brains of WT and MsrA KO mice were incubated with recombinant Nedd8 (FHN8) conjugates (8 µg) in PBS at 37 °C. After the designated time of incubation, equal volumes of 2× sample buffer were added and immediately heated at 100 °C for 5 min to terminate the reaction. The reaction solution of different time points was subjected to Western blot using mouse anti-His antibody (Qiagen, Hilden, Germany) as the primary antibody. The neddylation levels of the lowest molecular mass of the His-detected Nedd8 conjugates (L.M. Nedd8) were quantified by NIH Image-J program.

### 2.9. Jab1 Binding to P27 and P27 Expression in Brain

P27 from WT and MsrA KO brain extracts was immunoprecipitated with anti-P27 and the corresponding immunoprecipitates were probed with anti-Jab1 antibody by Western blot analyses.

## 3. Results

### 3.1. MsrA Interacting Proteins Identified by Yeast Two-Hybrid (Y2H)

MsrA interacting proteins of the *Homo sapiens* proteome were identified by a yeast-two hybrid (Y2H) approach. Using MsrA as the “bait” and human brain proteins as the “prey”, 36 hits to protein sequences were identified and are summarized in Table 1. The most prominent prey hits were Jab1 (CSN5) (*n* = 19), cyclin-I (*n* = 6), and succinate dehydrogenase [ubiquinone] iron-sulfur subunit (*n* = 6). These prominent hits were recurring suggesting specificity in the selection. In addition, five unique hits were identified, i.e., only one copy. The high number of hits obtained for Jab1, and the involvement of MsrA and Jab1 in Ub/Ub-like systems, prompted us to focus on this MsrA interacting target (Jab1).

### 3.2. GST-MsrA “Pull-Down” of Jab1

**Figure 2:**
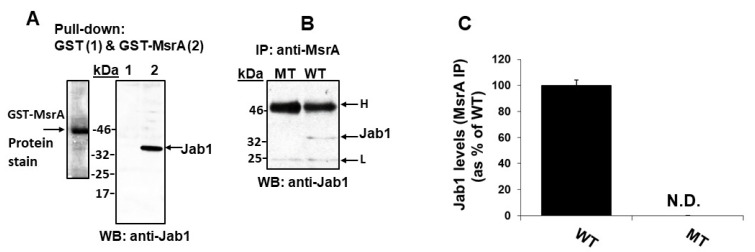
Interaction between MsrA and Jab1 in mouse brain extracts. (**A**) Pull-down experiments in which recombinant glutathione-*S*-transferase-MsrA (GST-MsrA) fused protein is used as a “bait” for binding of brain proteins. (**B)** Immunoprecipitation (IP) experiments using mouse brain extracts and anti-MsrA antibody (IP antibody), followed by Western blot analysis using mouse anti-Jab1 primary antibody. **C.** Quantification of the Jab1 band detected by Western blot analysis in Panel B, by using the NIH Image-J program. N.D, not detected; kDa, molecular mass markers in kilo-Dalton units; WT, wild-type mouse; MT, *MsrA* KO mouse; L, light chain of mouse immunoglobulin detected in the extract; H, heavy chain of mouse immunoglobulin detected in the extract. Protein stain, Coomassie blue for protein staining. This experiment was repeated at least 3 times and one set of data is represented in this figure.

To further probe the interaction of MsrA and Jab1, brain extracts were analyzed for pull-down of Jab1 using GST-MsrA as ‘bait’. MsrA was fused to glutathione-S-transferase (GST) and then bound to glutathione coated Sepharose resin. Thus, MsrA protein could be used as the “bait”, while GST resin alone served as a control (the relevant procedures are described in Section 2). Following gel-electrophoresis of the pulled-down proteins, one lane containing the GST-MsrA protein was stained for protein with Coomassie blue stain to observe the intensity of the major GST-MsrA protein band and the other minor bound proteins (Figure 2A). Additional gel-separated protein lanes were subjected to Western blot analysis using anti-Jab1 antibody as the primary antibody. As shown in Figure 2A, only the lane harboring the GST-MsrA showed a signal corresponding to Jab1, while the lane harboring GST alone showed no reaction. Thus, MsrA and Jab1 appeared to interact in a complex based on analysis by GST-MsrA pull down as well as Y2H.

### 3.3. Jab1 Co-Immunoprecipitates with MsrA

To further analyze this MsrA-Jab1 association, WT, and MsrA KO mouse brain extracts were subjected to immunoprecipitation using the anti-MsrA antibody. The resulting immunoprecipitates were analyzed for the presence of Jab1 by Western blotting using an anti-Jab1 antibody as the primary antibody. As this primary antibody was mouse generated, reactions with the mouse immunoglobulin light (L) and heavy (H) chains were anticipated and observed in both sample types (Figure 2B). However, a protein band specific to the WT mouse extract (and not in the MsrA KO mouse) was detected that migrated at a molecular mass comparable to Jab1 (Figure 2B), thus, providing further evidence that MsrA and Jab1 associate in a complex.

### 3.4. In Vivo Met Oxidation of Jab1 in Mouse Brain

**Figure 3:**
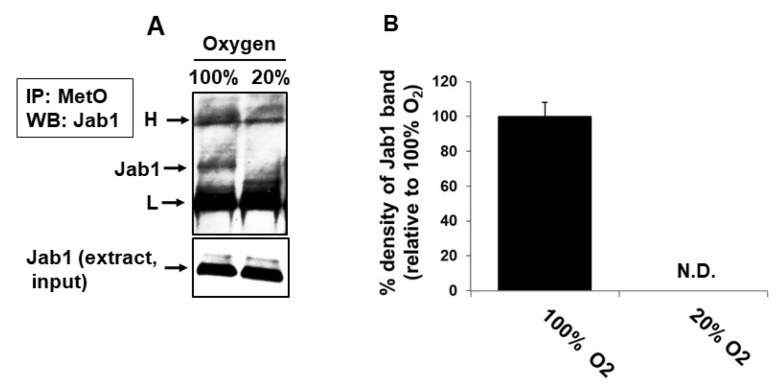
Met oxidation of Jab1 in mice exposed to hyperoxia (100% oxygen). WT mice (6 months old, *n* = 5) were exposed to hyperoxia for 24 h. Thereafter, the mice were euthanized and brain extracts were made in PBS as described under Section 2. (**A**) Immunoprecipitation with rabbit anti-MetO antibody were performed using 500µg of extracted protein from each brain, followed by Western blot analyses probed with the primary mouse anti-Jab1 antibody (see details under Section 2). (**B**) Quantification of the Jab1 band detected by Western blot analysis in Panel A, by using the NIH Image-J program. N.D, not detected; H, heavy chain of brain mouse IgG; L, light chain of brain mouse IgG. A representative blot is shown.

To determine whether Jab1 is susceptible to methionine oxidation in the brain, we exposed 6-month-old WT mice (*n* = 5 males) to normoxia (20% oxygen) or hyperoxia (100% oxygen) for 24 h as previously described [4] and then probed for MetO-Jab1. As shown in Figure 3, following the 24 h of hyperoxia, Jab1 contained MetO residues, as judged by the MetO-immunoprecipitation and subsequent anti-Jab1 Western blot analysis. In comparison, no MetO-Jab1 was detected in the control mice (*n* = 5 males) (Figure 3). These observations suggest that Jab1 is prone to methionine oxidation upon exposure of the animal to hyperoxic conditions [4].

### 3.5. MsrA-Mediated Deneddylase Activity of Jab1 on Cul-1

**Figure 4:**
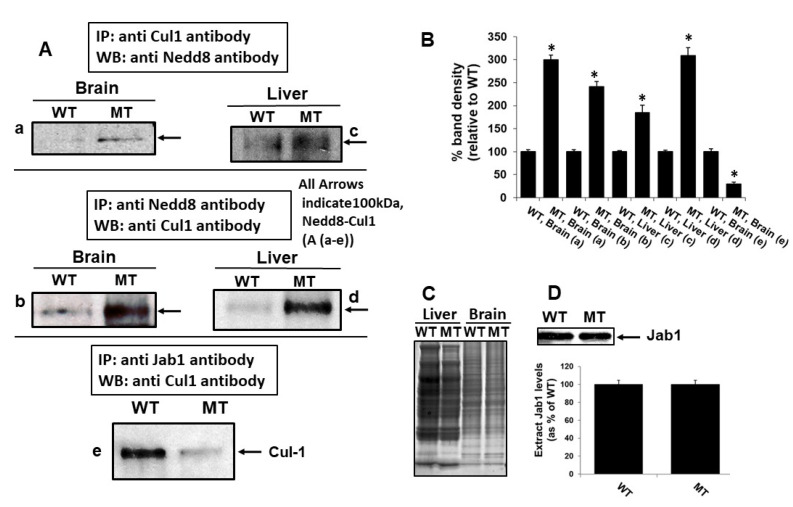
In vivo regulation of Jab1 activity by MsrA through monitoring Cul-1 neddylation levels in mouse brain and liver extracts. (**A**), a–d. Mouse extracts from 6 months old mice (*n* = 3) were made in PBS and in the presence of protease inhibitors cocktail (Sigma-Aldrich). Immunoprecipitation (IP) experiments were performed on brain and liver extracts (500 µg of protein per extract) using either anti-Cul-1 antibody or anti-Nedd8 antibody followed by Western blot (WB) analyses using anti-Nedd8 antibody or anti-Cul-1 antibody as the primary antibody, respectively. Ae. In a unique experiment, Jab1 was used as a “fishing” probe in an IP experiment using brain extracts, followed by Western blot analysis using anti-Cul-1antibody. Only the 100-kDa neddylated Cul-1 protein was identified due to the specific pull-down of the neddylated form of Cul-1 by Jab1. (**B**) Quantification of each band identified by Western blot analyses described in Panel (**A**) a–e, as determined by using the NIH Image-J program. All the observed differences in the observed band levels between the WT and MT pairs were statistically significant as judged by student *t*-test analysis (* *p* < 0.01, *n* = 3 per strain). (**C**) Loading controls for the liver and brain protein levels, following Coomassie blue staining (to confirm the use of equal amount of proteins from each mouse strain per tissue in the IP experiments. WT, wild type; M, MsrA KO. (**D**) Western blot analysis of the mouse brain extracts using anti Jab1 antibody as the primary antibody and quantified by the NIH Image-J program. WT, wild type; MT, *MsrA* KO. Arrows shown in (**A**) a–e indicate the position of the neddylated 100-kDa Cul-1 (deneddylated form runs as ~90 kDa protein, not detected). The shown WB and Coomassie blue staining experiments are representatives of three independent experiments.

To investigate the possibility that MsrA activates Jab1 function, we followed the abundance of deneddylated Cul-1 (a substrate of Jab1) as function of the presence or absence of MsrA in mouse brain and liver extracts. The added use of liver extracts was followed since MsrA is highly expressed in this organ; this approach also enabled us to investigate whether the effect of MsrA on Jab1 function was general in nature. To achieve this goal, proteins in both extract-types were immunoprecipitated with anti-Cul-1 antibody and then probed with anti-Nedd8 by Western blot analysis and vise-versa. As shown in Figure 4A (a,b), compared to WT brain, MsrA KO brain showed higher level of neddylated Cul-1. Higher Cul-1 neddylation level was also observed in MsrA KO liver extracts compared to WT (Figure 4A (c,d). Higher neddylation levels of Cul-1 in the MsrA KO brain and liver suggested that the Jab1 deneddylation activity was impaired in the absence of MsrA. This phenomenon was also in agreement with our hypothesis that MsrA reduction of specific MetO residue/s of Jab1 lead to increase of Jab1 activity, as measured by deneddylation of Cul-1. In the absence of MsrA (i.e., as demonstrated in the MsrA KO tissue extracts) the activity of Jab1 was compromised presumably due to its enhanced Met oxidation, resulting in more neddylation (or less deneddylation) of Cul-1.

### 3.6. MsrA Impact on Jab1 Binding to Cul-1

An active Jab1 presumably binds to the Nedd8 moiety of Cul-1 prior to removing this Ub-like modification from Cul1 (or any other target protein). Thus, we used the anti-Jab1 antibody in an IP experiment as a “fishing” probe to isolate protein complexes of neddylated Cul-1 bound to Jab1 from biological extracts and determine whether MsrA affected this binding. Indeed, following this approach, the WT brain showed much higher neddylated Cul-1 than the *MsrA* KO (MT), as judged by anti-Cul-1 Western blot analysis of samples IP with anti-Jab1 antibody (Figure 4A (e)). These data suggest that Jab1 binds Nedd8 on Cul-1 more effectively in the presence of MsrA, supporting the hypothesis stated above and suggesting that MetO-Jab1 is inactive in this binding. We then performed another Western blot analysis to investigate whether Jab1 levels changed in the *MsrA* KO compared with the WT mouse brain extracts. The results showed that the presence/absence of MsrA had no effect on the level of Jab1 (Figure 4D). Similar results were obtained using liver extracts (data not shown). These data indirectly support our hypothesis, that MsrA regulates Jab1 deneddylase activity through post-translational protein modifications (i.e., reduction of MetO-Jab1 to Jab1 forms) and not through alteration of Jab1 expression levels.

### 3.7. MsrA Impact on Jab1 Deneddylation Activity

**Figure 5:**
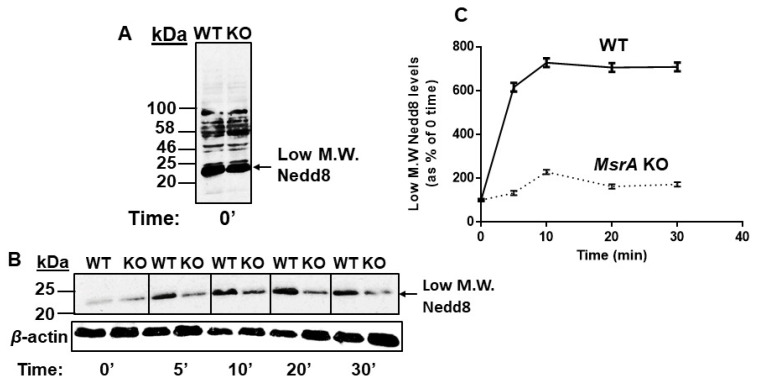
MsrA alters the kinetics of Jab1 deneddylase activity in vitro. (A) Western blot pattern of control group (time 0′). Total protein neddylation and Nedd8 levels are shown using the primary anti-His antibody. We focused on lowest molecular weight Nedd8 positive bands (Low M.W.Nedd8) as they represent the best end-product of the Nedd8 conjugates through the deneddylation process. (**B**) Quantification of lowest molecular weight Nedd8 positive bands at different time points following incubation with WT and MsrA KO mouse brain extracts (*n* = 3, females, 6 months old). (**C**) Time curve of lowest molecular weight Nedd8 levels. All data were normalized to 0 min time. The shown WB are representatives of three repeated experiments. WT, Wild-type; KO, *MsrA* knockout; The difference between the two animal groups was statistically significant from the 5–30 min time points (*t*-test, *p* < 0.001).

To examine the impact of MsrA on Jab1 deneddylation activity, equal amounts of brain extracted protein of WT and MsrA KO mice were incubated with Nedd8 (Flag-His-Nedd8, FHN8) conjugates at 37 °C. After the designated time of incubation, the reactions were terminated and subjected to Western blot analysis using mouse anti-His antibody (that recognizes FHN8). The neddylation levels of the lowest molecular mass of the His-detected Nedd8 conjugates (L.M.Nedd8) were quantified by NIH Image-J program. The rationale for this focused imaging was that the accumulation of L.M.Nedd8 would increase as a function of the incubation time due to the Jab1 activity. Indeed, at time zero, the neddylation levels of L.M.Nedd8 in the presence of WT and MsrA KO were identical (Figure 5A,B). However, the MsrA KO group exhibited much lower levels of released L.M.Nedd8 compared to the WT as the incubation time progressed. The deneddylation level of WT strain rapidly reached the peak after 10 min of incubation time that was much higher than the same peak observed for the MsrA KO strain (Figure 5A,B). This large difference in the deneddylase activity between the two strains remained up to the end of the incubation time (30 min). It is noteworthy, that the higher molecular Nedd8 conjugate levels also decreased proportionally to the increase of the Low M.W. Nedd8 levels (data not shown). Accordingly, the MsrA KO brain is suggested to exhibit much more Met-oxidized forms of Jab1 protein (MetO-Jab1) that are inactive compared with the WT brain. This phenomenon is in agreement with our hypothesis that lack of MsrA compromises the deneddylase activity of Jab1.

### 3.8. MsrA-Dependent Jab1 Binding to and Expression of P27

**Figure 6:**
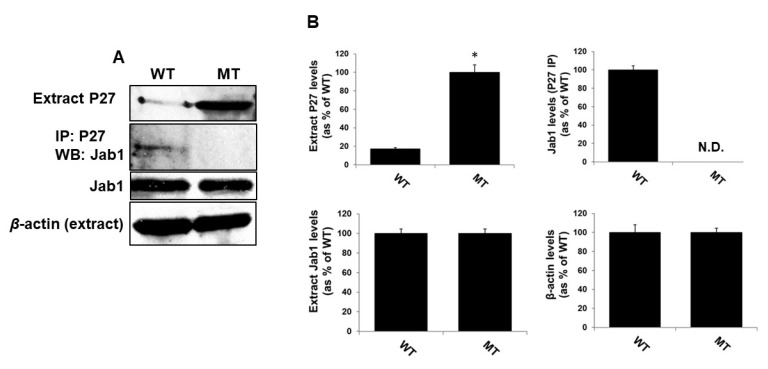
P27 binding to Jab1 and expression is mediated by MsrA. Mouse extracts were made in PBS and in the presence of protease inhibitors cocktail (Sigma-Aldrich, St. Louis, MO, USA) (6 months old mice, *n* = 3). (**A**) Immunoprecipitation (IP) experiments were performed on brain extracts (500 µg of protein per extract) using anti-P27 antibody followed by Western blot (WB) analyses using anti-Jab1 antibody as the primary antibody. WB analyses using the specific primary and suitable secondary antibody for each probed protein determined the expression levels of P27, Jab1, and β-actin (loading control). (**B**). Quantification of the each detected protein-band in the Western blot analyses described in Panel A, by using the NIH Image-J program. N.D, not detected; The same extracts used for the experiments performed in Figure 4 were used here. Western blot analysis for determining Jab1 and P27 expression levels in these brain extracts were analyzed using the same gel. Thus, for clarity reasons, the data shown for Jab1 expression in these extracts was duplicated from Figure 4D.

One of the functions of Jab1 is to bind P27 and to facilitate its translocation from the nucleus to the cytosol for Ub-dependent degradation. We propose that this function of Jab1 is strengthened by the function of MsrA. Here, we provide the first supportive evidence for this possibility by immunoprecipitation of P27 from WT and MsrA KO brain extracts and probing the P27 immunoprecipitates for Jab1 by applying Western blot analyses. As shown in Figure 6, the expression level of P27 was much lower in the WT compared with the MsrA KO brain, suggesting enhanced degradation and stability of P27 in WT and MsrA KO mice, respectively. Furthermore, Jab1 was detected in the immunoprecipitates of P27 in the WT brain but was not detected in the MsrA KO (Figure 6). This observation suggests that the increased binding of Jab1 to P27 is indeed mediated by the MsrA’s effect on Jab1; that in-turn causes an increase of P27 degradation in the WT brain (as suggested by the dramatic decrease of P27 protein expression in the WT strain). These observations strongly support the general idea that Jab1 function is compromised by the level of oxidized MetO residue/s that accumulate on this protein in the absence of MsrA. The levels of Jab1 protein were similar in both mouse strains, suggesting that the observed changes in Jab1-P27 complex levels are not due to a difference in Jab1 expression in the two mouse strains (Figure 6).

## 4. Discussion

In the studies described in this report, we showed that MsrA interacts with brain Jab1. We first demonstrated this interaction through Y2H screening (Table 1), and this interaction was validated by GST pull-down and immunoprecipitation experiments (Figure 2). Methionine oxidation of Jab1 was much greater in mouse brain following exposure to hyperoxia compared with normoxia, strengthening the hypothesis that Jab1 Met residues are oxidized under oxidative stress conditions in vivo (Figure 3). Furthermore, the ability of MsrA to increase Jab1 function (presumably through the reduction of critical MetO residue/s of Jab1) was further supported by the increase of deneddylation of Cul-1 in WT in comparison to *MsrA* KO brains (Figure 4). The positive effect of MsrA on the deneddylase activity of Jab1 was also monitored in vitro, using our novel recombinant Nedd8 conjugates as substrate (Figure 5).

In addition to its deneddylase activity, Jab1 is known for its function as a modulator of P27 degradation by binding to P27 in the nucleus and facilitating its translocation to the cytosol for Ub-dependent degradation [34]. Indeed, this function of Jab1 was positively affected by the presence of MsrA in the brain, as the binding of Jab1 to P27 was mostly detected in the WT brains, while a higher expression of P27 was observed in the MsrA KO compared with the WT brain (Figure 6).

**Figure 7:**
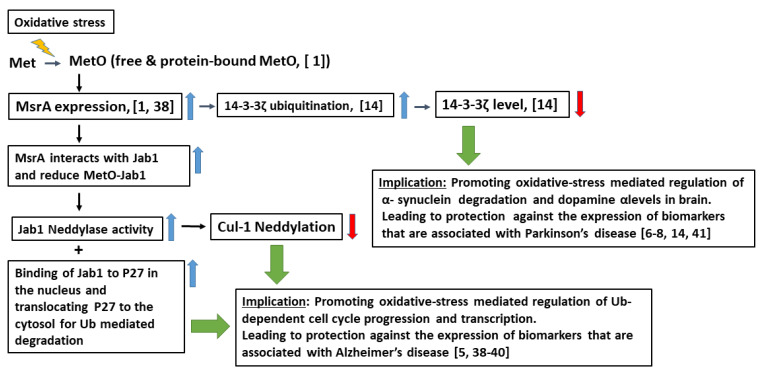
A summary of the proposed events and their link to neurodegeneration diseases. The details of the figure are presented under the Section 4. The blue arrows indicate an “upregulation” and the red arrows indicate a “downregulation” phenomenon.

Overall, the following scenario of events is suggested to occur in the brain upon oxidative stress conditions and are summarized in Figure 7. Briefly, due to enhanced oxidative stress, an elevated level of MetO (both as protein-bound and free MetO) is produced, causing an upregulation of MsrA through signal transduction pathways [1,37]. Consequently, the binding of MsrA to MetO-Jab1 is increased, leading to an activation of two major functions of Jab1: (a) binding to P27 and facilitating P27 degradation via the Ub-system, and (b) increasing the deneddylase activity of Jab1 towards neddylated proteins, in general, and of neddylated Cul-1, in particular. The sum of these events is expected to protect against oxidative-stress mediated abnormalities that are associated with Ub-dependent cell cycle progression and transcription. The overall implication of these MsrA-dependent phenomena is abolishing the expression of biomarkers that are associated with neurodegenerative diseases, such as AD [5,38,39,40] and Parkinson’s disease (PD; through an enhanced ubiquitination of 14-3-3 ζ [6,7,8,14,41]).

Our recent novel discovery demonstrates that MsrA is involved in the regulation of Ub-like modification of proteins in *Archaea* and ubiquitination of 14-3-3 ζ in the mouse brain [14,15]. Thus, the interaction of MsrA with Jab1, which is playing a role in the ubiquitination and neddylation processes, is an additional intriguing component. Establishing the role of MsrA as a positive regulator of Jab1 will have implications for the general function of MsrA in mediating protein ubiquitination/neddylation, as well as to enhancing cell survival, especially in neurodegenerative diseases. For example, under enhanced oxidative stress conditions, cells may be vulnerable to reactive oxygen species-mediated damage. To circumvent this situation, the cells upregulate MsrA to rescue and/or increase Jab1 function through the reduction of its MetO residue/s. In turn, the protected Jab1 activity can prevent the degradation of Cullin-RING E3 ligases by the Ub/Ub-like dependent degradation pathways. Furthermore, the function of MsrA prevents the accumulation of undesirable levels of P27 that are better regulated via the MetO-reduced forms of Jab1 that drive P27 for Ub-dependent degradation. These phenomena have implications for cell survival under oxidative stress conditions that are commonly present in aging, age-associated diseases, such as neurodegenerative diseases (e.g., AD and PD).

AD is characterized by the degeneration of neuronal populations mainly in the hippocampus, leading to progressive neurological malfunction [42]. Up-to-date, the causes and pathogenesis of AD are not clear. Accumulative evidence suggests that dysregulation of several cell cycle factors is contributing to the pathophysiology of AD [43]. Consequently, the neurons of the AD brain display various cell cycle markers prior to degeneration [44]. Accordingly, cell cycle proteins are suggested to play an important and vital role in neuronal cell death. For example, cell cycle proteins are required for the neuronal death induced by amyloid-beta, the major peptide component of senile plaques. Alteration in the expression of cell cycle factors has also been linked to apoptosis of neurons. With respect to AD specifically, both P27 and phosphorylated P27 showed elevated levels in the cytoplasm of vulnerable neuronal populations in AD disease vs. control subjects [39]. Importantly, the phosphorylated P27 showed association with tau-positive neurofibrillary pathology, including neurofibrillary tangles, dystrophic neurites, and neuropil threads [39]. Likewise, higher levels of P27 were observed in a mouse model of AD [38]. Thus, these data suggest that dysregulation of the cell cycle plays a crucial role in the pathogenesis of AD that may be controlled by the interaction of Jab1 and MsrA. Cell cycle plays an important role in the development of cancer and relatively higher levels of Jab1 are associated with malignancy in specific cancerous cells [45]. The role of MsrA in regulating Jab1 function in cancer is yet to be determined.

For example, according to the acquired knowledge on MsrA role in the above processes, developing or identifying small molecules that can penetrate the brain and upregulate MsrA could be beneficial for protecting against the development of AD [19]. In addition to the need to acquire new knowledge on the functional role of MsrA within the neddylation and ubiquitination pathways, determining the sequence region of Jab1 that binds MsrA and enables this interaction will be important. Availability of such data will provide fundamental information that can be used in follow up structure-function studies. For example, protein crystallization experiments, aimed to define the structural complex of these two proteins, can expand our knowledge with respect to these proteins’ interaction and hint on the possibilities to enhance their binding to each other. Accordingly, determining the conditions that affect the complex stability of MsrA-Jab1 may be used as potential tools to modulate Jab1 activity when it is strongly associated with the development and progression of a specific disease such as AD.

## 5. Conclusions

**Figure 8:**
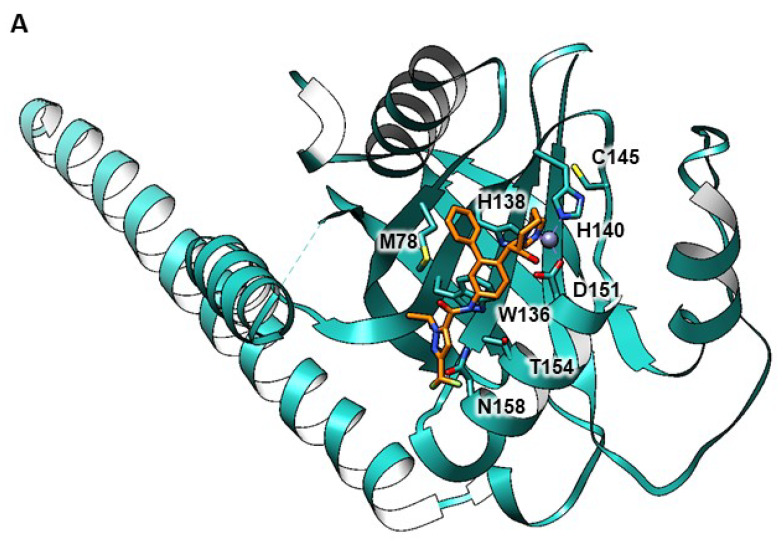

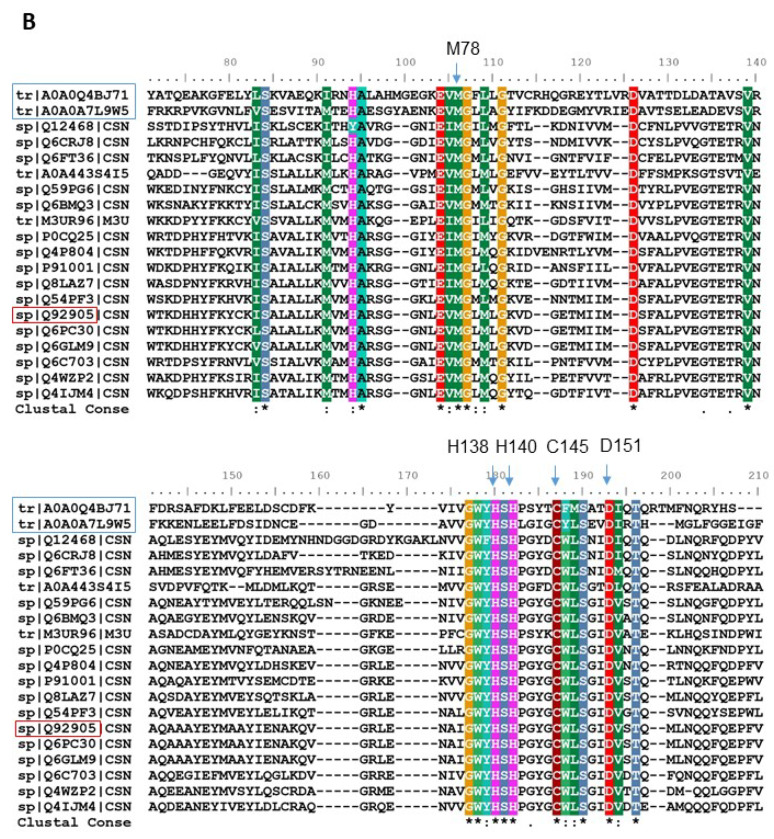
Proposed importance of Met78 in regulation of Jab1 function by MsrA. (**A**) Jab1 (blue ribbon) in complex with inhibitor (orange) revealing the close proximity of methionine 78 (M78) to the substrate binding cleft of the Zn^2+^ metalloprotease active site. Stick diagram is used to indicate the inhibitor (CSN5i-3), residues that coordinate Zn^2+^ (His138, His140 and D151) and other residues in the active site region (M78, T154, N158, W136 and C145). Crystal structure [PDB: 5JOG, PMID: 27774986] [46] was visualized using Chimeria v1.14 [PMID: 15264254] [47]. (**B**) Multiple amino acid sequence alignment of the central domain of representative Jab1 homologs selected from diverse organisms. Coloring and * or: indicates regions of 100% identity or similarity, respectively. Residues that coordinate Zn^2+^ (His138, His140 and D151) and other residues in the active site region that may be susceptible to oxidation (M78 and C145) are indicated by down arrows. Protein identifier indicated on left with *Homo sapiens* denoted by a red box and Archaea indicated by blue boxes (see Table S1 for complete listing of the proteins in the alignment).

The studies and data presented in this article clearly advocate for the independent and combined importance of Jab1 and MsrA in regulating cellular regulation posttranslational modifications. Both methionine oxidation and neddylation of proteins are well described as key protein modifiers that can affect protein structure and function. A major question that remains and needs further investigation is: how does MsrA interaction with Jab1 affect Jab1 activity and the resulting deneddylation levels of proteins? One possibility is that the Jab1 active site has Met residues susceptible to oxidation and inactivation that can be repaired by MsrA. Consistent with this possibility, the Jab1 active site has a Met78 residue within the putative substrate-binding pocket that is in close proximity to the active site formed by residues that coordinate a catalytic Zn^2+^ ion (Figure 8A). Furthermore, the Met78 residue of Jab1 is evolutionary conserved across a diverse spectrum of organisms from *H. sapiens* to methanogenic archaea suggesting it has an important biological role (Figure 8B). Understanding the relationships between MsrA and Jab1-dependent protein deneddylation and P27 degradation will provide important knowledge on Jab1 regulation and its mechanism of action. Future new data is expected to fill up the gap in knowledge related to the function of Jab1 within the Ub/Ub-like system and related diseases. In addition, overexpression of MsrA in the brain, through novel treatments, is anticipated to protect against the expression of biomarkers associated with neurodegenerative diseases.

## Figures and Tables

**Table 1:** Msr type A enzyme (MsrA) interacting proteins identified by yeast two-hybrid system analysis.

No. of Hits	GenBank No.	Description [Homo Sapiens]	Met	Cys
19 ^a^	NP006828	COP9 signalosome complex subunit 5 (Jab1/CSN5)	3.9% ^b^	1.2%
6	NP006826.1	Cyclin-I (Cyc-1)	2.9%	2.9%
6	NP002991.2	Succinate dehydrogenase [ubiquinone] iron-sulfur subunit, mitochondrial precursor	3.6%	5.0%
1	NP689854.2	AT-rich interactive domain-containing protein 2	1.9%	1.6%
1	NP004757.1	Coatomer subunit β’	2.0%	1.7%
1	NP055093.2	Heat shock 70 kDa protein 4L	2.7%	2.1%
1	NP005678.3	Phenylalanyl-tRNA synthetase β chain	2.0%	1.9%
1	NP001171895.1	WD repeat-containing protein 44 isoform 3	1.7%	1.0%

^a^ Hits that mapped to protein sequence (36 total) were identified from 18 positive clones from the initial SD-LTHA selection and others from the SD-LTH selection. Forty-three hits were identified using BlastN program (nucleotide) and 36 hits were identified using BlastX program (protein). ^b^ % total of deduced protein sequence.

## Supplementary Materials

The following are available online at https://www.mdpi.com/2076-3921/9/5/452/s1, Table S1: Uniprot-numbers-Jab1-homologs.

## Abbreviations

Jab1: c-Jun activation domain-binding protein-1; P27, cyclin-dependent kinase inhibitor 1B; Cul-1, Cullin-1; Msr, methionine sulfoxide reductase; MsrA, A-type Msr; MetO, methionine sulfoxide; Nedd8, neural precursor cell expressed developmentally down-regulated 8; Ub, ubiquitin; NAE, Nedd8 activating enzyme; NCE, Nedd8 conjugating enzyme; RBX1, RING-box protein 1; CRL, Cullin/RING ubiquitin ligase; SCF, Skp1/Cul-1/F-box.
